# Pulsed Electromagnetic Fields Modulate Inflammatory and Tenogenic Responses in Human Tenocytes: Insights from Acute and Prolonged Inflammation Models

**DOI:** 10.3390/cells15171529

**Published:** 2026-08-25

**Authors:** Michela Maria Taiana, Paola De Luca, Giulio Grieco, Enrico Ragni, Simona Salati, Antonio Marmotti, Valerio Pascale, Laura de Girolamo

**Affiliations:** 1 IRCCS Ospedale Galeazzi Sant’Ambrogio, Laboratorio di Biotecnologie Applicate all’Ortopedia, 20157 Milan, Italy; michelamaria.taiana@grupposandonato.it (M.M.T.); enrico.ragni@grupposanadonato.it (E.R.); laura.degirolamo@grupposandonato.it (L.d.G.); 2 Medical Division, IGEA, 41012 Carpi, Italy; s.salati@igeamedical.com; 3 Department of Orthopaedics and Traumatology, University of Turin, 10100 Turin, Italy; antonio.marmotti@inwind.it; 4 Molecular Biotechnology Center “Guido Tarone”, Department Molecular Biotechnology and Health Sciences, University of Turin, 10100 Turin, Italy; 5 IRCCS Ospedale Galeazzi Sant’Ambrogio, Ortopedia Clinicizzata, 20157 Milan, Italy; valerio.pascale@unimi.it; 6 Medicina e Chirurgia, Scienze Biomediche per la Salute, Università degli Studi di Milano, 20133 Milan, Italy

**Keywords:** tendinopathy, pulsed electromagnetic fields (PEMFs), human tenocytes, Interleukin-1β (IL-1β), inflammatory models

## Abstract

**Highlights:**

**What are the main findings?**

- PEMF stimulation promotes tenocyte proliferation across acute inflammatory models and enhances wound closure capacity, with PEMFs significantly improving wound closure in IL-1β-treated cells under acute low-dose inflammation.
- PEMF effects on inflammatory mediators, including IL-6, IL-8, GM-CSF, CCL2 and CCL5, vary according to the intensity and duration of the inflammatory stimulus.

**What is the implications of the main finding?**

- PEMFs are likely to support tenocyte regenerative behaviour during acute or mild inflammatory phases, suggesting that an evaluation of the inflammatory status is a critical determinant of treatment efficacy.

**Abstract:**

Tendinopathy is a prevalent musculoskeletal condition characterised by chronic inflammatory and degenerative changes. Pulsed electromagnetic fields (PEMFs) represent a promising biophysical therapeutic modality, yet their effects across different inflammatory states of tendinopathy remain poorly characterised. To evaluate PEMF biological effects on human tenocytes, three in vitro models differing in IL-1β dose and duration were tested: acute low-dose (0.1 ng/mL, 96 h), acute high-dose (1 ng/mL, 96 h), and prolonged (0.1 ng/mL, 9 days). At the transcriptional level, PEMFs significantly reduced *IL-6* and *IL-8* mRNA overexpression in the acute low-dose model and *CCL2* upregulation in the prolonged model. At the secretome level, PEMFs reduced GM-CSF and IL-8 secretion in the acute high-dose model, and suppressed CCL2 and CCL5 protein secretion in the prolonged model. MMP activity was not modulated by PEMFs in any condition. PEMFs consistently increased tenocyte proliferation across both the acute models. Finally, in wound healing assays, non-inflamed tenocytes exposed to PEMFs showed significantly enhanced wound closure compared to IL-1β-treated cells across all models; in the acute low-dose model, PEMFs also significantly improved wound closure in IL-1β-treated cells at an early timepoint, whereas no such effect was observed in cells exposed to high-dose or prolonged inflammation. PEMFs exert context-dependent effects, promoting healing primarily during acute or mild inflammation. These findings suggest that the inflammatory stage of tenocytes may influence PEMF responsiveness in vitro, highlighting the importance of considering this variable in the design of future clinical studies evaluating PEMF therapy for tendinopathy.

## 1. Introduction

Tendinopathy is a spectrum of chronic tendon disorders affecting both the general population and athletes, characterised by persistent pain, localised swelling, and impaired function. The condition accounts for approximately 30% of referrals to musculoskeletal practitioners and represents a substantial socioeconomic burden [1]. Although historically classified as a degenerative disorder driven by mechanical overload and failed healing, increasing evidence has reframed tendinopathy as a disease with a significant inflammatory component, involving complex crosstalk between structural, cellular, and molecular mechanisms [2,3]. The transition from healthy to pathological tendon is characterised by extracellular matrix (ECM) disorganisation, hypercellularity, and a profound phenotypic shift in tenocytes, which lose their tenogenic identity and acquire a catabolic and pro-inflammatory profile [4,5].

Interleukin-1β (IL-1β) has emerged as a pivotal mediator in tendinopathy pathogenesis. This pro-inflammatory cytokine is upregulated in diseased tendon tissue and promotes a catabolic cascade in tenocytes, including upregulation of cyclooxygenase-2 (COX2), prostaglandin E2 (PGE2), and matrix metalloproteinases (MMPs), alongside downregulation of critical tenogenic transcription factors such as scleraxis (SCX) [4,6]. Moreover, IL-1β perpetuates inflammatory cycles through activation of NF-κB and MAPK signalling pathways, and has been implicated in the loss of tendon stem/progenitor cell identity [7].

Current conservative and surgical treatments for tendinopathy demonstrate limited efficacy, underscoring the need for novel therapeutic strategies. Pulsed electromagnetic fields (PEMFs) represent a non-invasive biophysical intervention with established applications in bone healing and increasing evidence supporting their role in soft tissue repair. In a previous study, our group demonstrate that in a rat model of collagenase-induced Achilles tendinopathy, daily PEMF exposure improved tendon fibre organisation and restored physiological tissue architecture, particularly when applied during the mid-acute phase of disease [8]. PEMFs are thought to modulate cellular behaviour through interactions with membrane-associated receptors, particularly A_2A_ adenosine receptors, and the subsequent attenuation of pro-inflammatory signalling pathways [9,10]. In vitro studies in tenocytes have already demonstrated PEMF-mediated reductions in IL-6, IL-8, COX-2, and MMP expression, together with increased type I collagen synthesis [9,11,12].

Within the broader landscape of engineering strategies for musculoskeletal tissue regeneration, PEMFs represent a non-genetic approach to directing tenocyte behaviour through controlled physical stimulation, providing a complement to biomaterial and cell-based regenerative strategies.

However, a critical limitation of the existing studies is their reliance on single, acute inflammatory models, which fail to recapitulate the heterogeneity and the different stages of tendinopathy. Chronic tendinopathy encompasses distinct inflammatory phases, from acute reactive stages, characterised by rapid cytokine upregulation and acute ECM remodelling, to prolonged degenerative states associated with sustained low-grade inflammation and loss of tenogenic commitment, which may respond differently to biophysical stimulation [1,4]. No study to date has systematically compared PEMF effects across inflammatory models of varying dose and duration in human tenocytes.

The present study therefore aimed to comprehensively evaluate PEMF effects on human tenocytes using three distinct IL-1β-based inflammatory models encompassing inflammatory conditions ranging from acute to more sustained, prolonged stages of tendinopathy: an acute low-dose model, an acute high-dose model, and a prolonged model. PEMF effects were assessed in terms of gene expression profiles, secretion of inflammatory mediators, MMP activity, cell migration, and proliferation, with the overarching aim of defining the inflammatory context in which PEMF stimulation exerts the most relevant biological activity.

## 2. Materials and Methods

### 2.1. Cell Culture

Human tenocytes (TCs) were isolated from gracilis and semitendinosus tendon biopsies obtained from *n* = 4 consenting donors (all male; mean age 25.5 ± 6.2) undergoing anterior cruciate ligament reconstruction, in accordance with institutional ethical approval (“Caratterizzazione e valutazione del potenziale rigenerativo delle cellule progenitrici tessuto specifiche ottenute da tessuto muscoloscheletrici”, approval date 16 December 2020, registered under number 214/int/2020 for surgery room waste material). Tendon fragments were minced into 2–3 mm^2^ pieces and enzymatically digested with 0.3% type I collagenase (Worthington Biochemical Co., Lakewood, NJ, USA) for 16 h at 37 °C under continuous agitation. After centrifugation (376× *g*, 5 min at room temperature), cells were resuspended in low-glucose Dulbecco’s Modified Eagle Medium (LG-DMEM, Biowest, Nuaillé, France) containing L-Glutamine and sodium Pyruvate, supplemented with 10% foetal bovine serum (FBS), 1% Penicillin–Streptomycin (PSG; Life Technologies, Carlsbad, CA, USA), and 1% Fungizone (Life Technologies, Carlsbad, CA, USA), seeded at 5 × 10^3^ cells/cm^2^ and maintained at 37 °C in a humidified atmosphere containing 5% CO_2_. TCs were isolated by plastic adherence, expanded to passage 3, and subsequently used for all experimental analyses to ensure phenotypic consistency. Cells from each donor were processed and analysed separately, without pooling, yielding four independent biological replicates per experimental condition.

### 2.2. Inflammatory Stimulation with IL-1β and PEMF Treatment

**Scheme 1:**
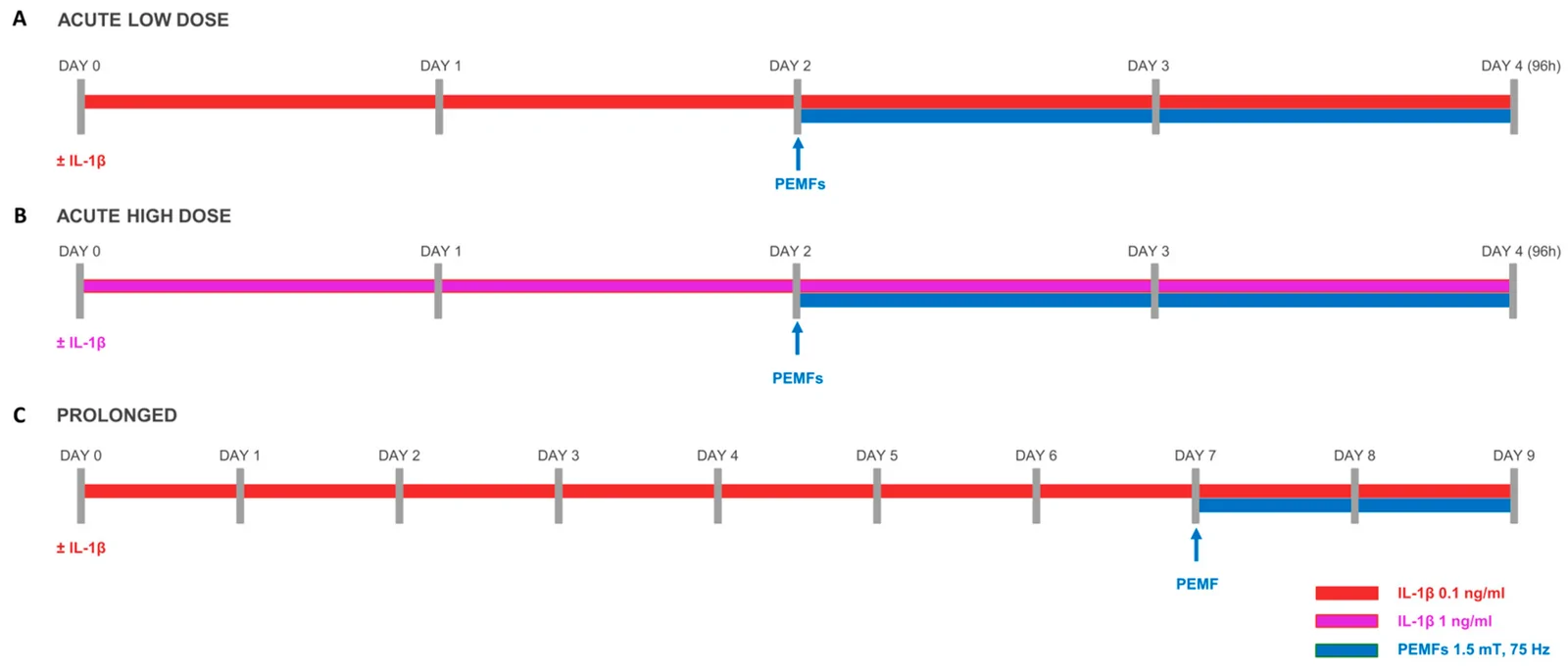
Experimental design for acute and prolonged inflammatory stimulation models. Cells were exposed to IL-1β and pulsed electromagnetic field (PEMF, 1.5 mT) stimulation according to two time-course protocols. In the acute model (96 h), IL-1β was administered at 0.1 ng/mL ((**A**): Low) or 1 ng/mL ((**B**): High) from Day 0, with the PEMF applied concomitantly from Day 2 for the remaining 48 h. In the prolonged model ((**C**): 9 days), cells were treated with 0.1 ng/mL IL-1β throughout, and the PEMF was co-administered from Day 7 for the final 48 h. Red and fuchsia bars indicate IL-1β stimulation; blue bars indicate PEMF treatment.

To model both acute and prolonged tendon inflammation, TCs were subjected to IL-1β stimulation according to two experimental protocols, as illustrated in Scheme 1. In the acute model, cells were cultured in DMEM control medium supplemented with IL-1β at either 0.1 ng/mL (acute low dose) or 1 ng/mL (acute high dose) for a total of 96 h. In the prolonged model, IL-1β was administered at 0.1 ng/mL for 9 consecutive days. The high-dose IL-1β condition (1 ng/mL) replicates the protocol previously used by our group to model tenocyte inflammation [10]. In both protocols, pulsed electromagnetic field (PEMF) stimulation was applied during the final 48 h, concurrently with the ongoing inflammatory stimulus (Scheme 1). PEMF stimulation was delivered using a pair of rectangular horizontal coils (18 cm × 13 cm; 1000 turns of copper wire each) positioned opposite each other inside a standard cell culture incubator. The coils were powered by a PEMF generator system (IGEA, Carpi, Italy) set at 1.5 mT, 75 Hz, trapezoidal waveform, pulse length 1.1 ms, and a 10% duty cycle. Culture flasks were placed at the central region between the coils, where field uniformity had been verified by the manufacturer. PEMF exposure was continuous throughout the treatment period at 37 °C. Non-PEMF-exposed control flasks were cultured in a separate incubator without the coil apparatus.

### 2.3. Gene Expression Analyses

Total RNA was extracted using the RNeasy^®^ Mini Kit (Qiagen, Venlo, The Netherlands) according to the manufacturer’s instructions, and cDNA was synthesised with the iScript™ cDNA Synthesis Kit (Bio-Rad Laboratories, Segrate, Italy). Gene expression of the following targets was quantified by real-time PCR using iTaq Universal SYBR Green Supermix (Bio-Rad Laboratories, Segrate, Italy) on the QuantStudio™ 12K Flex OpenArray^®^ Platform (QS12KFlex, Thermo Fisher Scientific, Waltham, MA, USA): *ACAN*, *ADAMTS*, *BDNF*, *CCL2*, *CCL5*, *CGRP*, *COL1*, *COL3*, *COX2*, *DCN*, *IDO*, *IL1B*, *IL1RN*, *IL6*, *IL8*, *MKX*, *MMP1*, *MMP3*, *NANOG*, *NGF*, *OCT4*, *PTGES2*, *SCX*, *SSEA1*, *SOX2*, *TAC1*, *TIMP1*, *TNC*, and *VEGF*. Reference gene stability was assessed across all experimental conditions by evaluating eight candidate housekeeping genes (*RPL13A*, *YWHAZ*, *B2M*, *GAPDH*, *ACTB*, *TBP*, *RPLP0*, and *18S*) using the RefFinder tool (https://www.heartcure.com.au/reffinder/) accessed on 13 May 2026, which integrates the geNorm, NormFinder, BestKeeper, and comparative ΔCt methods. *GAPDH* was identified as the most stable reference gene and was used for normalisation in all subsequent analyses. Primer sequences are reported in Table S3.

### 2.4. ELISAs

Concentrations of soluble cytokines (CCL2, CCL5, GM-CSF, IL-4, IL-6, IL-8, IL-10, IL-22, IL-23, and IL-28A) in culture supernatants were determined by ELISA using commercial kits from PeproTech (Hamburg, Germany) and Invitrogen (Carlsbad, CA, USA), following the manufacturers’ protocols. The specific detection ranges for PeproTech kits were 15–1000 pg/mL (IL-4/IL-8), 31–2000 pg/mL (IL-6), and 47–3000 pg/mL (IL-10). For Invitrogen kits, the ranges were 7–1000 pg/mL (CCL2), 6–750 pg/mL (GM-CSF), 8–1000 pg/mL (IL-22), 16–2000 pg/mL (IL-23), 15.6–1000 pg/mL (IL-28A), and 47–3000 pg/mL (CCL5). Data were acquired through a PerkinElmer Wallac Victor II plate reader (PerkinElmer, Waltham, MA, USA) and processed and analysed using MyAssays2.com platform (2026). To account for PEMF-induced differences in cell number across conditions, cytokine concentrations were normalised to cell number and expressed as amount per 10^6^ cells.

### 2.5. Metalloproteinase (MMP) Activity Assay

MMP activity levels were evaluated in culture media using the SensoLyte 520 Generic MMP Assay Kit (AnaSpec, Fremont, CA, USA). Following the manufacturer’s guidelines, pro-MMPs were converted to their active forms via incubation with 1 mM APMA at 37 °C for 3 h. Fluorescence signals (λex = 490 nm; λem = 520 nm) were then recorded utilising a plate reader (PerkinElmer, Waltham, MA, USA).

### 2.6. Proliferation, Viability and Wound Healing Assays

Cell proliferation and viability were assessed by fluorescence-based counting using Acridine Orange/DAPI solution (A13, ChemoMetec), according to the manufacturer’s instructions. Briefly, 10 µL of staining reagent was added to 190 µL of each cell suspension and analysed with a NucleoCounter^®^ NC-3000™ (ChemoMetec, Allerod, Denmark).

Wound healing capacity was evaluated by scratch assay. TCs were seeded at 5 × 10^3^ cells/cm^2^ in 24-well plates and, at the onset of PEMF stimulation (Day 2 for the acute model; Day 7 for the prolonged model), a cross-shaped scratch was introduced into the confluent monolayer using a sterile 20 µL pipette tip. Detached cells were removed by PBS washing and fresh medium was added. Images were acquired at T0 (immediately after scratching, 0 h), T1 (8 h), T2 (24 h), T3 (32 h), and T4 (48 h) using an inverted phase-contrast microscope. Wound closure was quantified with ImageJ free software (https://imagej.net/ij/) and expressed as percentage relative to T0: [(wound area T0 − wound area Tt)/wound area T0] × 100.

### 2.7. Statistical Analysis

All experiments were performed on *n* = 4 independent tendon donors, with cells from each donor processed and analysed separately (no pooling), yielding four independent biological replicates per condition. Technical replicates varied by assay and the mean of the technical replicates was calculated for each donor prior to statistical analysis. All analyses were conducted using GraphPad Prism v9.5.0 (GraphPad Software, San Diego, CA, USA). Data normality was evaluated via the Shapiro–Wilk test. Continuous data are expressed as mean ± SD. For ELISA, MMP activity, gene expression, and proliferation data, differences between groups were analysed by two-way ANOVA (matching across donors; factors: IL-1β dose and PEMF treatment) followed by Sidak’s multiple comparisons test. For wound healing data, a mixed-effects model analysis was applied, followed by Tukey’s multiple comparisons test within each timepoint. For repeated-measures designs, sphericity violations were addressed using the Greenhouse–Geisser correction. Statistical significance was defined as a two-tailed *p*-value < 0.05. Effect sizes (η^2^, expressed as % of total variation attributable to each source) for two-way ANOVA models are reported in Table S4; for CCL5 secretion, analysed using a mixed-effects model, F values are reported. Post hoc pairwise comparisons with 95% confidence intervals of the mean difference are reported in Table S5.

## 3. Results

### 3.1. PEMFs Promote Tenocyte Proliferation in Acute Inflammatory Models

**Figure 1:**
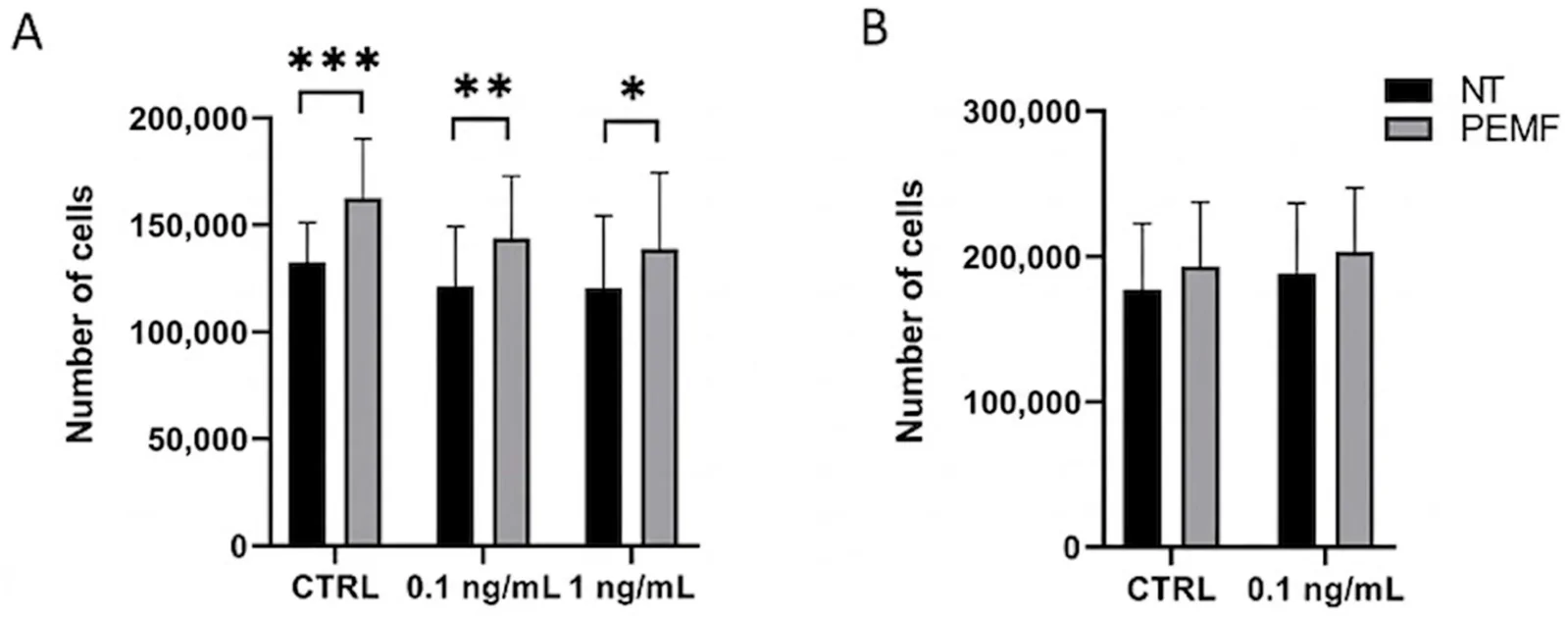
PEMFs increase tenocyte number in acute inflammatory models. Cell number, assessed by fluorescence-based counting under acute (**A**) and prolonged (**B**) inflammatory conditions. Cells were cultured in control medium (CTRL) or stimulated with IL-1β (0.1 ng/mL or 1 ng/mL) in the presence (PEMF) or absence (NT) of pulsed electromagnetic field stimulation. Data are presented as mean ± SD. Significant differences between NT and PEMF-treated groups are indicated as follows: * *p* < 0.05, ** *p* < 0.01, *** *p* < 0.001. No statistically significant differences were detected in the prolonged model (**B**). NT: non-treated; PEMF: pulsed electromagnetic field.

PEMF stimulation robustly and significantly increased tenocyte proliferation in the acute model across all conditions, including non-inflamed controls (*p* < 0.001), low-dose IL-1β (*p* < 0.01), and high-dose IL-1β (*p* < 0.05) (Figure 1A). At the omnibus level, PEMFs accounted for a significant proportion of variance in the acute model (η^2^ = 16.4%, *p* = 0.0099), whereas IL-1β concentration alone did not reach significance (η^2^ = 7.2%, *p* = 0.14; Table S4). In the prolonged model, although a similar trend was observed, no significant difference in proliferation was observed between PEMF-treated and untreated cells in either control or IL-1β-stimulated conditions (Figure 1B). Cell viability remained consistently high (96–98%) across all conditions and timepoints, with no significant differences between NT and PEMF-treated groups in any model (Table S2), indicating that neither IL-1β nor PEMF exposure induced overt cytotoxicity in this system and that the observed increase in cell number reflects genuine proliferation rather than differential cell survival.

### 3.2. PEMFs Enhance Wound Closure in Non-Inflamed Tenocytes and Improve Wound Closure in IL-1β-Treated Cells Under Acute Low-Dose Inflammation

Wound healing assays were performed across all three inflammatory models at four sequential timepoints (T1–T4).

**Figure 2:**
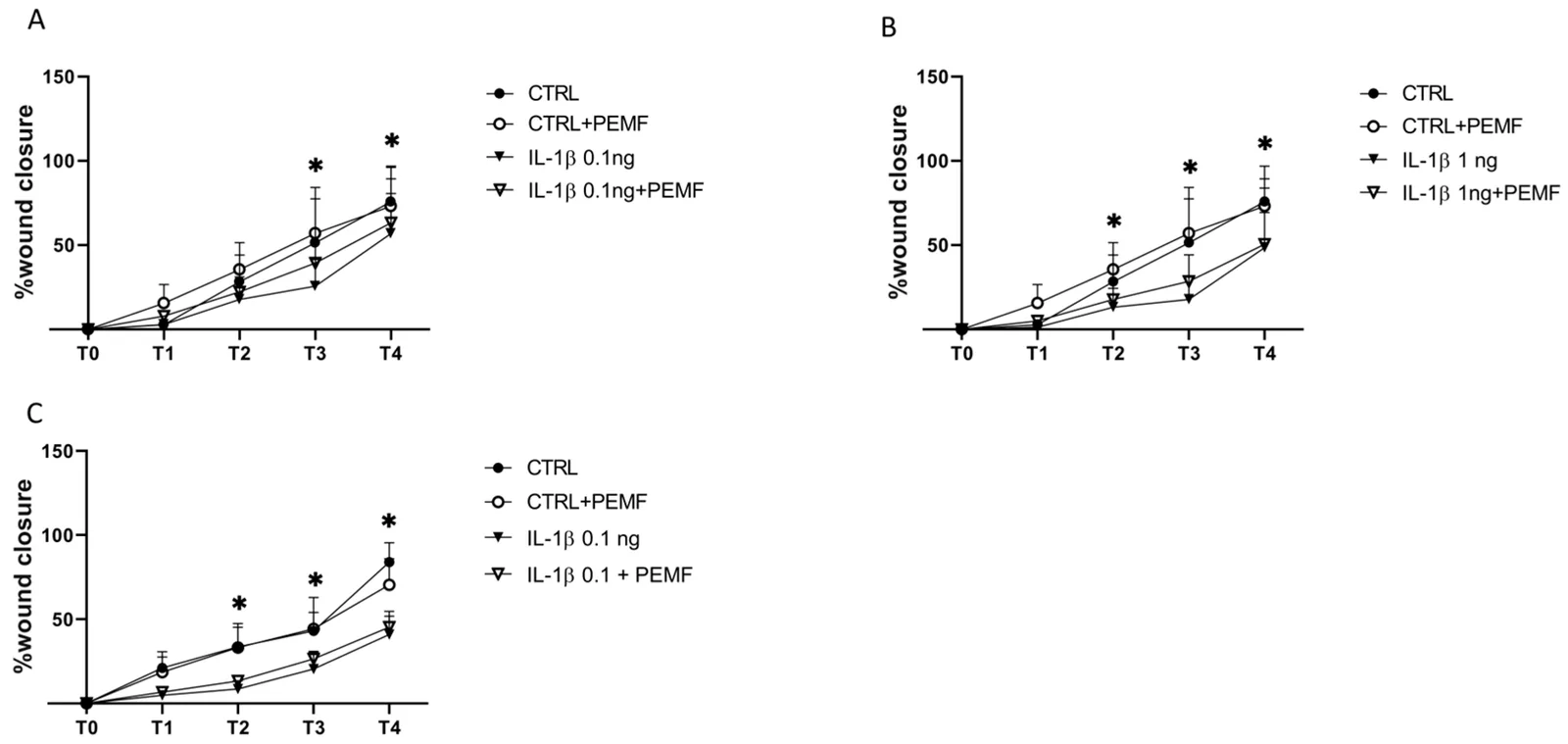
PEMFs enhance wound closure in non-inflamed tenocytes and improve wound closure in IL-1β-treated cells under acute low-dose inflammation. Scratch-wound healing assays evaluated over 4 sequential timepoints (T1–T4) across three experimental conditions: acute low-dose (**A**), acute high-dose (**B**), and prolonged (**C**) inflammatory models. Quantitative analysis of the percentage of wound closure is plotted for non-treated controls (CTRL), PEMF-stimulated controls (CTRL + PEMF), IL-1β-stimulated cells, and IL-1β cells treated with PEMFs (IL-1β + PEMF). Data represent mean ± SD. * *p* < 0.05 indicates statistically significant differences between groups as detailed in the text.

In the acute low model (Figure 2A), direct comparisons showed that PEMFs significantly improved wound closure in IL-1β-treated cells relative to IL-1β alone at T3 (*p* < 0.05), while no significant effect of PEMFs was observed in non-inflamed controls at this timepoint (CTRL vs. CTRL + PEMF, ns). Non-inflamed PEMF-treated cells (CTRL + PEMF) also showed significantly greater wound closure compared to IL-1β-treated cells at T3 (*p* < 0.05). At T4, wound closure was significantly lower in IL-1β-treated cells compared to CTRL (*p* < 0.05); at this later timepoint, PEMFs no longer significantly affected wound closure in either non-inflamed (CTRL vs. CTRL + PEMF, ns) or IL-1β-treated cells (IL-1β vs. IL-1β + PEMF, ns).

In the acute high model (Figure 2B), CTRL + PEMF showed significantly greater wound closure compared to IL-1β-treated cells at T2, T3, and T4 (*p* < 0.05 for all). However, direct comparisons showed no significant effect of PEMFs on either non-inflamed controls (CTRL vs. CTRL + PEMF) or IL-1β-treated cells (IL-1β vs. IL-1β + PEMF) at any timepoint, indicating that PEMFs did not significantly alter wound closure within either baseline condition at this higher inflammatory dose.

In the prolonged model (Figure 2C), CTRL + PEMF showed again significantly greater wound closure compared to IL-1β-treated cells at T2 and T3 (*p* < 0.05). At T4, only CTRL retained significantly higher wound closure than IL-1β-treated cells (*p* < 0.05). As in the other models, direct comparisons showed no significant effect of PEMFs on either non-inflamed controls or IL-1β-treated cells at any timepoint in the prolonged model.

### 3.3. PEMFs Selectively Modulate Inflammatory Gene Expression Depending on Tendinopathy Model

IL-1β induced a broad inflammatory–catabolic response across all conditions, including upregulation of *IL6*, *IL8*, *CCL2*, *COX2*, *IDO1*, *MMP1*, and *MMP3*, together with downregulation of the tenogenic transcription factor *SCX*. The prolonged model further exhibited a significant decrease in *MKX*, *ACAN*, and *CD44* expression, consistent with a deeper degenerative phenotype. A comprehensive summary is provided in Table S1.

**Figure 3:**
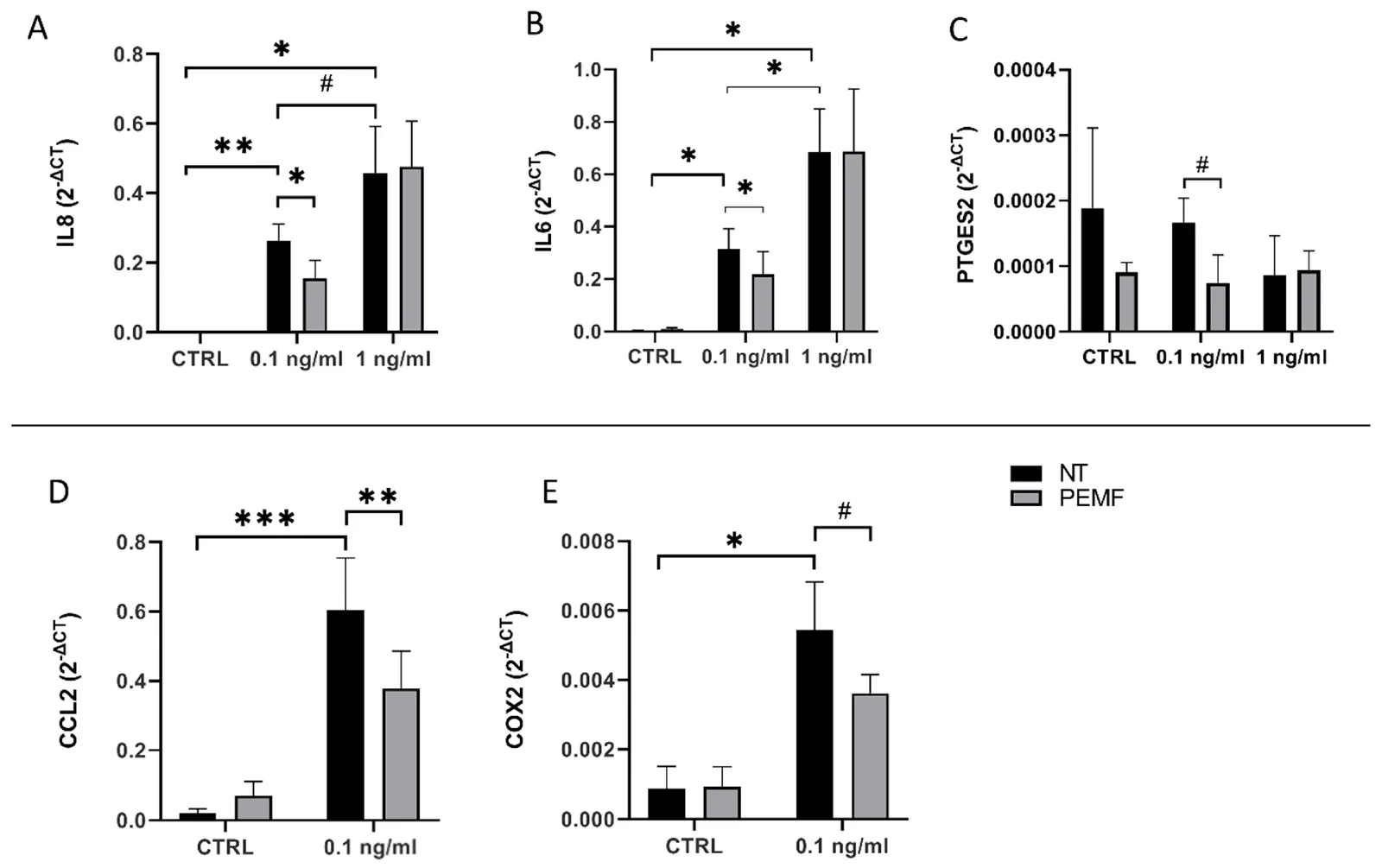
PEMFs selectively modulate inflammatory gene expression at the transcriptional level depending on model context. Real-time PCR (qPCR) analysis of target gene expression under acute (**A**–**C**) and prolonged (**D**,**E**) inflammatory conditions induced by IL-1β. Relative mRNA expression levels (2^−∆CT^) are shown for *IL8* (**A**), *IL6* (**B**), and *PTGES2* (**C**) in the acute model, and for *CCL2* (**D**) and *COX2* (**E**) in the prolonged model. Data are presented as mean ± SD. Significant differences between groups are indicated as follows: * *p* < 0.05, ** *p* < 0.01, *** *p* < 0.001; # *p* < 0.1 indicates a statistical trend. NT: non-treated; PEMF: pulsed electromagnetic field.

At the transcriptional level, PEMFs demonstrated targeted anti-inflammatory rescue activity in a model-dependent manner. Notably, PEMFs significantly rescued the overexpression of both *IL6* and *IL8* mRNA in the acute low model (*p* < 0.05). In the prolonged inflammatory model, PEMFs achieved significant rescue of *CCL2* overexpression (*p* < 0.01). Consistent with this, PEMFs accounted for a larger proportion of variance in *CCL2* expression than IL-1β itself (η^2^ = 78.8% vs. 7.5%; Table S4). *COX2* was significantly upregulated by IL-1β across all models and showed a trend toward PEMF-mediated downregulation in the prolonged model (*p* = 0.08). *PTGES2* also showed a tendency toward downregulation following PEMF stimulation in the acute low model, though IL-1β did not induce significant upregulation of this gene (Figure 3).

In the acute low condition, *SCX* and *TNC* showed a tendency toward further reduction following PEMF treatment; as these effects did not reach statistical significance and occurred against a background of already-compromised tenogenic commitment driven by IL-1β, their biological relevance remains to be confirmed.

The remaining IL-1β-regulated genes, including *COL1*, *COL3*, *CCL5*, *IDO*, *ICAM1*, *MMP1*, *MMP3*, and *VEGF*, were not significantly modulated by PEMFs in any condition (Table S1).

### 3.4. PEMFs Selectively Modulate Cytokine Secretion Depending on Tendinopathy Model

ELISA analysis of conditioned supernatants revealed model-dependent effects of PEMFs on cytokine secretion. Among the cytokines assessed, GM-CSF, IL-6, CCL2, CCL5, and IL-8 were detected, whereas IL-4 showed no significant differences across experimental conditions. IL-10, IL-22, IL-23, and IL-28A remained below the assay detection threshold in all conditions and were therefore excluded from further analysis. IL-1β significantly induced GM-CSF, IL-6, CCL2, CCL5, and IL-8 under at least some experimental conditions, although not uniformly across models. Specifically, IL-8 and IL-6 were significantly induced only in the acute high-dose model, whereas CCL5 was significantly induced only in the acute low-dose model. In contrast, GM-CSF, and CCL2 were significantly increased following both acute IL-1β stimulations.

PEMFs affected GM-CSF, with a significant reduction in the acute high-dose model (*p* < 0.01) and a trend toward reduction in the acute low-dose and prolonged models.

For IL-8, PEMFs reduced it significantly in the acute high-dose model only (*p* < 0.001).

**Figure 4:**
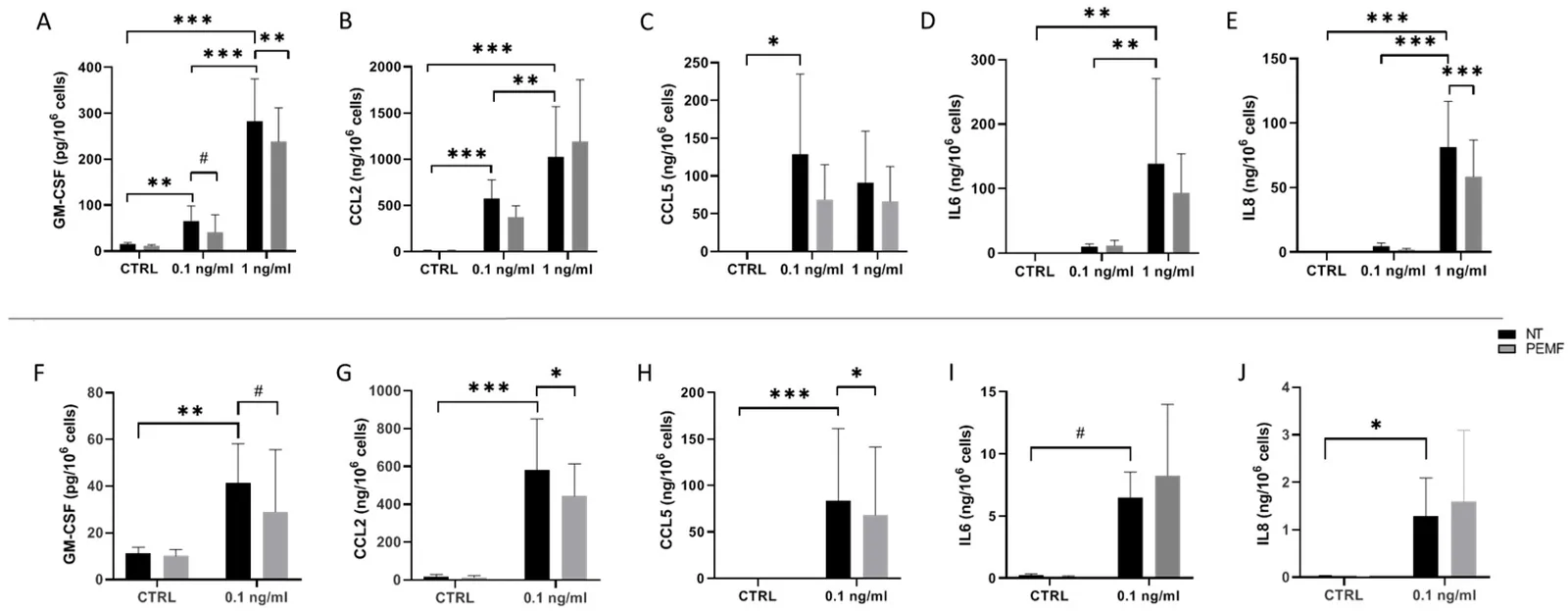
PEMFs selectively modulate cytokine and chemokine secretion depending on model context. ELISA quantification of protein levels in conditioned supernatants, normalised to cell number (per 10^6^ cells), under acute (**A**–**E**) and prolonged (**F**–**J**) inflammatory conditions. General response to IL-1β stimulation and the modulatory effect of PEMF treatment are shown for GM-CSF (**A**,**F**), CCL2 (**B**,**G**), CCL5 (**C**,**H**), IL-6 (**D**,**I**), and IL-8 (**E**,**J**). Data are expressed as mean ± SD. Significant differences are indicated as follows: * *p* < 0.05, ** *p* < 0.01, *** *p* < 0.001 for comparison between groups; # *p* < 0.1 indicates a statistical trend. NT: non-treated; PEMF: pulsed electromagnetic field.

PEMFs significantly reduced CCL2 and CCL5 secretion in the prolonged model (*p* < 0.05) (Figure 4).

### 3.5. PEMFs Do Not Affect IL-1β-Induced MMP Activity

**Figure 5:**
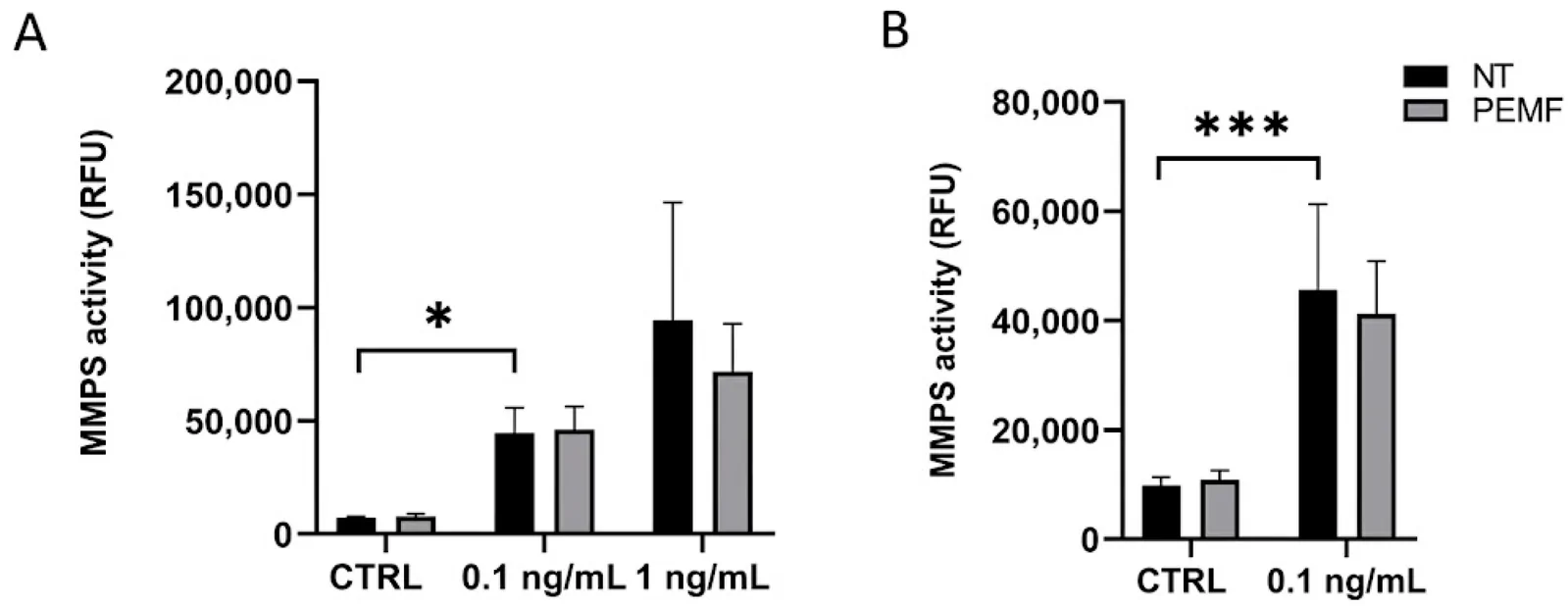
PEMFs do not modulate IL-1β-induced total MMP activity. Fluorometric substrate assay quantification of total matrix metalloproteinase (MMP) activity in conditioned supernatants under acute (**A**) and prolonged (**B**) inflammatory conditions. Relative enzymatic activity is expressed as Relative Fluorescence Units (RFU). Data are presented as mean ± SD. Significant increases induced by IL-1β compared to control (CTRL) groups are indicated by asterisks (* *p* < 0.05, *** *p* < 0.001). No statistically significant differences were observed between NT and PEMF-treated samples in any condition. NT: non-treated; PEMF: pulsed electromagnetic field.

MMP activity assays, performed following APMA-mediated activation of pro-MMPs to assess total activatable MMP activity, demonstrated a significant increase in response to both acute low-dose IL-1β exposure (*p* < 0.05) (Figure 5A) and prolonged low-dose exposure (*p* < 0.001) (Figure 5B). In the acute high-dose condition, a trend toward increased MMP activity was observed, though this did not reach statistical significance due to inter-sample variability. PEMF treatment did not significantly modulate MMP activity in any experimental condition in the current static monolayer system.

## 4. Discussion

This study provides a systematic characterisation of PEMF effects on human tenocytes across three IL-1β-based inflammatory models of differing intensity and duration. Our findings demonstrate that PEMFs exert selective, context-dependent biological effects with anti-inflammatory and pro-regenerative activity predominantly during acute, low-grade inflammatory conditions. This observation has important methodological and clinical implications, and may partly explain the heterogeneous outcomes reported in the clinical literature on PEMF therapy in tendinopathy. Notably, this phase-dependent responsiveness is consistent with in vivo evidence from a rat model of Achilles tendinopathy, in which PEMF exposure was more effective in restoring physiological tendon architecture when applied during the mid-acute phase of disease compared to earlier or later stages [8], suggesting that the inflammatory/reparative stage of the tissue may be a key determinant of PEMF efficacy across experimental systems.

The selective transcriptional effects exerted by PEMFs suggest that biophysical stimulation can modulate specific pathways according to the inflammatory state.

Specifically, the rescue of *IL-6* and *IL-8* in the acute low model is consistent with previous reports describing PEMF-mediated suppression of NF-κB and MAPK signalling in different experimental settings [9]. The lack of this effect in the acute high model suggests that increasing inflammatory burden progressively reduces the capacity of PEMFs alone to counteract IL-1β-driven signalling. Conversely, *CCL2* rescue in the prolonged model may reflect a distinct mechanism operative under sustained low-grade inflammatory conditions, possibly involving adenosine receptor-mediated pathways [10]. Consistent with the transcriptional data, PEMFs accounted for the largest share of variance in *CCL2* expression among the sources tested, supporting a robust and reproducible modulatory effect on this specific target, even in a context where IL-1β dominates overall variance across most inflammatory readouts.

The protein secretome analysis revealed that GM-CSF emerged as one of the most consistently PEMF-responsive secreted mediators. In fact, GM-CSF was significantly elevated by IL-1β in a dose-dependent manner, while PEMF significantly reduced its secretion in the acute high-dose model, with a trend toward reduction in the acute low-dose and prolonged models. This is particularly noteworthy given the central role of GM-CSF in amplifying local inflammation through the activation and differentiation of monocytes and macrophages, and its capacity to drive further production of IL-6, IL-1, and matrix-degrading proteases [13]. IL-8 secretion was significantly reduced by PEMFs at the higher acute dose.

CCL2 and CCL5 followed a similar pattern across models. Both chemokines were significantly induced by IL-1β in the acute model (CCL2 at both doses, CCL5 at the lower dose only), but PEMFs did not significantly affect their secretion under acute conditions. In the prolonged model, however, the PEMF significantly reduced both CCL2 and CCL5 secretion, suggesting that its immunomodulatory activity may become more evident under sustained, low-grade inflammatory stimulation, potentially limiting the chemokine-driven recruitment of monocytes that characterises prolonged inflammatory states. Nevertheless, as the present in vitro system comprised tenocyte monocultures without immune cell co-culture, these paracrine and chemotactic interpretations remain speculative and warrant direct functional validation in future non-autonomous systems.

The absence of statistically significant PEMF-mediated MMP modulation in our in vitro models should be interpreted in the context of the well-recognised limitations of static monolayer models in recapitulating tendon pathophysiology. Our system lacks mechanical loading, three-dimensional ECM architecture, vascular interactions, and immune cell crosstalk, all of which critically shape MMP regulation in vivo and may be required to fully unmask biophysical effects on matrix remodelling. Notably, other in vitro studies using IL-1β-stimulated human tenocytes have reported significant PEMF-mediated suppression of MMP-1, MMP-2, and MMP-3 [9], a discrepancy that may reflect differences in PEMF dosimetry (field intensity, frequency, duty cycle), exposure duration, or cell donor variability between the two experimental settings. Importantly, the absence of MMP modulation in vitro cannot be equated with clinical inefficacy, given the multiple in vivo mechanisms through which PEMFs are known to exert their effects.

Tenocyte viability, monitored across all conditions, remained consistently high (96–98%) and was not significantly affected by IL-1β or PEMF exposure in any model. The proliferative response of tenocytes to PEMFs was consistently observed in the acute model across all conditions regardless of inflammatory status, suggesting a direct pro-survival response to biophysical stimulation that operates independently of the IL-1β cascade, possibly mediated by A_2A_ adenosine receptor upregulation [10,12] or modulation of growth factor receptor signalling. In the prolonged model, a consistent trend toward increased proliferation was observed, suggesting that this activity may extend to more established inflammatory states, pending confirmation with larger sample sizes. The most parsimonious interpretation is that this could represent a transient proliferative-expansion phase that precedes re-differentiation, consistent with established models of tissue repair biology even if longitudinal and protein-level studies are required to confirm this.

The wound healing analysis provides compelling evidence of PEMF-mediated conditioning of tenocyte wound closure capacity. Since no anti-mitotic agent was employed, the observed wound closure may reflect the combined contribution of PEMF-enhanced proliferation and migration. The consistent advantage in wound closure observed in CTRL + PEMF cells across all three models suggests that the PEMF acts primarily by enhancing the functional competence of tenocytes that retain cytoskeletal plasticity and proliferative responsiveness before severe inflammatory dysfunction becomes established. Notably, PEMFs significantly improved wound closure in IL-1β-treated cells relative to IL-1β alone at T3, before the IL-1β-induced deficit became statistically established (evident only from T4 onward), at which point PEMFs no longer showed a significant effect. This temporal pattern suggests that PEMF-mediated functional benefit may be most readily detectable in the earlier phase of inflammatory stimulation, before cumulative IL-1β exposure imposes a more dominant suppressive effect on cytoskeletal dynamics and focal adhesion signalling, consistent with the hypothesis of a potential narrowing window of cellular responsiveness as inflammatory burden progresses, which requires further validation. These findings suggest the existence of an inflammatory threshold beyond which PEMF-mediated functional recovery becomes progressively limited, possibly reflecting the dominant suppressive effect of IL-1β on cytoskeletal dynamics and focal adhesion signalling [14], although this mechanism was not directly investigated in the present study. Overall, these findings suggest an in vitro window of cellular responsiveness to PEMFs, whereby the biological responsiveness of tendon cells progressively declines with increasing inflammatory burden. Whether this in vitro observation reflects a clinically relevant window of responsiveness remains a hypothesis to be tested in future in vivo and clinical studies.

Taken together, these findings define a biological profile for PEMFs in human tenocytes characterised by consistent pro-proliferative activity, context-dependent anti-inflammatory modulation at both transcriptional and secretome levels, absence of functional MMP suppression in the current experimental system, and a conditioning effect on wound closure capacity restricted to non-inflamed or mildly inflamed cells.

Several limitations of the present study must be acknowledged. The relatively small sample size and the intrinsic biological variability among human tendon donors may limit the generalisability of the observed trends, particularly for effects that approached but did not reach statistical significance. As already mentioned, the static two-dimensional monolayer system lacks the physiological ECM architecture, dynamic mechanical loading, vascular interactions, and immune cell crosstalk that critically shape tenocyte behaviour and MMP regulation in vivo, and the 9-day prolonged model should not be equated with clinically defined chronic tendinopathy, which develops over months. No formal a priori power calculation was performed. The sample size (*n* = 4 donors) was chosen based on comparable published in vitro studies of PEMF effects on primary human tenocytes, and on the practical constraints associated with the availability of primary human tendon tissue. However, it remains modest, which limits statistical power and the generalisability of the present findings, particularly with regard to effects of smaller magnitude. A further limitation is the absence of a sham-PEMF control, as untreated cells were maintained in a separate standard incubator. Although this setup does not fully exclude non-specific effects related to device handling, a sham-exposed control will be incorporated in future studies to strengthen attribution of the observed effects to PEMF exposure. Additionally, cytokines for which concentrations fell below the assay detection threshold in all samples (IL-10, IL-22, IL-23, and IL-28A) were excluded from quantitative statistical comparison and are reported descriptively as undetectable, rather than being imputed; this conservative approach may underestimate low-magnitude PEMF effects on these mediators. The absence of immune cells is a particularly relevant constraint, given the central role of macrophage polarisation in orchestrating both the inflammatory and resolution phases of tendon pathology. It is therefore possible that PEMF effects on matrix remodelling and tenocyte phenotype under physiologically relevant conditions differ substantially from those observed here. Furthermore, intracellular signalling pathways were not directly investigated at the protein level (e.g., via Western blot or immunostaining); while secreted levels of selected inflammatory mediators were assessed by ELISA, direct assessment of pathway activation was not performed, limiting mechanistic conclusions regarding the intracellular molecular targets of PEMFs. While cell viability was systematically monitored and remained unaffected across all conditions, dedicated assays for apoptosis and ion channel dynamics were not performed, and represent relevant mechanistic layers for future investigation. More broadly, mechanistic interpretations of intracellular signalling pathways and the contribution of immune cell contributions remain speculative and are proposed as hypotheses for future investigation, rather than as demonstrated findings.

Future studies incorporating three-dimensional bioreactor cultures, mechanical stimulation, and immune cell co-culture systems are required to validate and extend these findings, and to determine whether the hypothesised window of cellular responsiveness identified in vitro translates to clinically meaningful outcomes across the different stages of tendinopathy.

## 5. Conclusions

Pulsed electromagnetic fields (PEMFs) have been shown to exert biological effects on human tenocytes, promoting cell proliferation and wound closure, particularly during the acute stages of the inflammatory response. PEMFs also selectively modulate key inflammatory mediators, at both the transcriptional and secretome levels. Notably, these effects are dependent on the intensity and duration of the inflammatory stimulus, being more pronounced under acute or mild inflammatory conditions, which may represent a window of greater biological responsiveness that may be relevant for future translational studies. Collectively, although with the intrinsic limitation of an in vitro model, these findings support the use of PEMFs as an adjunctive regenerative strategy during the acute or mild inflammatory phases of tendinopathy, supporting their potential as a non-invasive biophysical tool within the broader landscape of regenerative engineering strategies for musculoskeletal tissue repair. Furthermore, this study highlights the critical impact of inflammatory model selection on the observed therapeutic response, underscoring the need for standardised, multi-model approaches in preclinical tendon research and providing a rationale for further investigating stage-specific treatment strategies in future in vivo and clinical studies.

## Data Availability

The raw data are available at the link https://osf.io/8sphz/overview?view_only=0e3297d2856a4d07a7780c6454d50da6 accessed on 30 June 2026.

## Supplementary Materials

The following supporting information can be downloaded at https://www.mdpi.com/article/10.3390/cells15171529/s1: Table S1: Summary of IL-1β-induced gene expression changes and PEMF modulation across inflammatory models. Table S2: Cell viability across experimental conditions. Table S3: Primer sequences used for real-time PCR analysis. Table S4: Effect sizes (η^2^, % of total variation) and *p*-values from two-way ANOVA for each outcome measure. Table S5: Post hoc pairwise comparisons (mean difference, 95% CI, adjusted *p*-value) for each outcome measure.

## Abbreviations

The following abbreviations are used in this manuscript:

ACAN	Aggrecan
ADAMTS	A Disintegrin and Metalloproteinase with Thrombospondin Motifs
APC	Allophycocyanin
APMA	4-Aminophenylmercuric Acetate
BDNF	Brain-Derived Neurotrophic Factor
CCL2	C-C Motif Chemokine Ligand 2
CCL5	C-C Motif Chemokine Ligand 5
CGRP	Calcitonin Gene-Related Peptide
COL1	Collagen Type I
COL3	Collagen Type III
COX2	Cyclooxygenase-2
CTRL	Control
DAPI	4′,6-Diamidino-2-Phenylindole
DMEM	Dulbecco’s Modified Eagle Medium
DCN	Decorin
ECM	Extracellular Matrix
EDTA	Ethylenediaminetetraacetic Acid
ELISA	Enzyme-Linked Immunosorbent Assay
FBS	Foetal Bovine Serum
GAPDH	Glyceraldehyde 3-Phosphate Dehydrogenase
GM-CSF	Granulocyte-Macrophage Colony-Stimulating Factor
IDO/IDO1	Indoleamine 2,3-Dioxygenase 1
IL-1β	Interleukin-1β
IL1RN	Interleukin 1 Receptor Antagonist
IL	Interleukin (IL-4, IL-6, IL-8, IL-10, IL-22, IL-23, IL-28A)
IQR	Interquartile Range
MAPK	Mitogen-Activated Protein Kinase
MKX	Mohawk Homeobox
MMP	Matrix Metalloproteinase (MMP1, MMP3)
NF-κB	Nuclear Factor Kappa B
NGF	Nerve Growth Factor
PBS	Phosphate-Buffered Saline
PEMF	Pulsed Electromagnetic Field
PGE2	Prostaglandin E2
PRP	Platelet-Rich Plasma
PSG	Penicillin–Streptomycin–Glutamine
PTGES2	Prostaglandin E Synthase 2
RANTES	Regulated on Activation, Normal T Cell Expressed and Secreted
SCX	Scleraxis
SOX2	SRY-Box Transcription Factor 2
SSEA1	Stage-Specific Embryonic Antigen 1
TAC1	Tachykinin Precursor 1
TCs	Tenocytes
TIMP1	Tissue Inhibitor of Metalloproteinases 1
TNC	Tenascin C
VEGF	Vascular Endothelial Growth Factor
