# Supplementary material for: Pulsed Electromagnetic Fields Modulate Inflammatory and Tenogenic Responses in Human Tenocytes: Insights from Acute and Prolonged Inflammation Models
Source: Cells. 2026 Aug 25;15(17):1529. doi: 10.3390/cells15171529 (PMC13565733; doi:10.3390/cells15171529)
Supplement: Supplementary file 1 [file cells-15-01529-s001.zip › Table_S1.pdf]

Supplementary Table S1. Summary of IL-1β-induced gene expression changes and PEMF modulation across inflammatory models.

Gene	Early Low (IL-1β)	Early High (IL-1β)	Prolonged (IL-1β)	PEMF Effect
ACAN	↓	↓	↓ *tend*	–
ADAMTS	↓ *tend*	–	–	–
CCL2	↑	↑	↑	Rescue ↓ in Prolonged ($p<0.05$)
CCL5	↑	–	–	–
CD44	–	–	↓	–
COL1	↑ *tend*	–	–	–
COL3	↑	↑	–	–
COX2	↑	↑	↑	Partial rescue ↓ in Prolonged (tend.)
CXCL10	–	–	↑	–
FN1	–	–	↑ *tend*	–
ICAM1	↑	↑	–	–
IDO1	↑	↑	↑	–
IL-1	↑ *tend*	–	–	–
IL-1RN	–	↑ *tend*	–	–
IL-6	↑	↑	↑	Rescue ↓ in Early Low ($p<0.05$)
IL-8	↑	↑	↑	Rescue ↓ in Early Low ($p<0.05$)
IL-10	–	↓ *tend*	–	–
MKX	–	–	↓	–
MMP1	↑	↑	↑	–
MMP3	↑ *tend*	–	↑	–
SCX	↓	↓	–	Concordant trend ↓ in Early Low (tend.)
SSEA1	–	–	↓	–
TNC	–	–	–	Concordant trend ↓ in Early Low (tend.)
VEGF	–	↑	–	–

↑ upregulation; ↓ downregulation; – no significant change; *tend* = tendency ($p<0.10$). ***Bold italic*** = gene name. Green highlight = statistically significant PEMF rescue ($p<0.05$); yellow = trend toward PEMF partial rescue; red = trend toward adverse PEMF effect.
