# Supplementary material for: Pulsed Electromagnetic Fields Modulate Inflammatory and Tenogenic Responses in Human Tenocytes: Insights from Acute and Prolonged Inflammation Models
Source: Cells. 2026 Aug 25;15(17):1529. doi: 10.3390/cells15171529 (PMC13565733; doi:10.3390/cells15171529)
Supplement: Supplementary file 1 [file cells-15-01529-s001.zip › Table_S2.pdf]

Supplementary Table S2. Cell viability across experimental conditions. Cell viability (%) assessed by Acridine Orange/DAPI fluorescence staining (NucleoCounter NC-3000) at the endpoint of each experimental protocol (96 h for acute models, 9 days for the prolonged model), in non-treated (NT) and PEMF-treated tenocytes under control and IL-1β-stimulated conditions. Data from n = 4 tendon donors are reported individually and as mean ± SD.

Condition	Donor 1	Donor 2	Donor 3	Donor 4	Mean ± SD
CTRL early	98.45	96.10	97.65	97.95	97.54 ± 1.01
CTRL+PEMF early	94.85	97.40	98.35	98.35	97.24 ± 1.65
IL-1β 0.1 ng/ml early	97.50	98.50	95.55	96.75	97.08 ± 1.24
IL-1β 0.1 ng/ml + PEMF early	92.95	98.50	98.75	96.70	96.72 ± 2.68
IL-1β 1 ng/mL early	96.60	97.25	96.45	99.15	97.36 ± 1.24
IL-1β 1 ng/mL + PEMF early	94.80	97.40	96.15	97.85	96.55 ± 1.37
CTRL prolonged	98.20	96.55	96.75	97.00	97.12 ± 0.74
CTRL+PEMF prolonged	97.45	97.45	98.25	99.275	98.11 ± 0.87
IL-1β 0.1 ng/mL 9d prolonged	98.00	99.05	93.95	98.00	97.25 ± 2.25
IL-1β 0.1 ng/mL + PEMF prolonged	96.55	98.85	96.975	98.575	97.74 ± 1.14
