# Supplementary material for: Pulsed Electromagnetic Fields Modulate Inflammatory and Tenogenic Responses in Human Tenocytes: Insights from Acute and Prolonged Inflammation Models
Source: Cells. 2026 Aug 25;15(17):1529. doi: 10.3390/cells15171529 (PMC13565733; doi:10.3390/cells15171529)
Supplement: Supplementary file 1 [file cells-15-01529-s001.zip › Table_S3.pdf]

Supplementary Table S3. Primer sequences used for quantitative real-time PCR.

Gene	Forward primer	Reverse primer
ACAN	AGATGGACACCCCATGCAAT	GGCTGTGCCCTTTTTACACG
ADAMTS1	TGGCGTCAATGCTTTCCAAC	TGTCCATCAAACATTCCCCATGA
BDNF	TGTCTGTGGCCTTGTTCTCTC	ATTTACAATAGGCTTCTGATGCG
CCL2	GCAATCAATGCCCCAGTCAC	CTTGAAGATCACAGCCTTCTTTGG
CCL5	GGTACCATGAAGGTCTCCGC	GGTGTCCGAGGAATATGGGG
CGRP	CCCAGAAGAGAGCCTGTGACA	CTTCACCACACCCCCTGATC
COL1A1	GTGGCCCAGAAGAACTGGTA	GCCGCCATACTCGAACTGGA
COL3A1	CTTCTCTCCAGCCGAGCTTC	TGTGTTTCGTGCAACCATCC
COX2	CAAATTGCTGGCAGGGTTGC	AGGGCTTCAGCATAAAGCGT
DCN	CATCCAGGTTGTCTACCTTCATA	CAGAGCGCACGTAGACACAT
IDO1	GCTAAAGGCGCTGTTGGAAA	TTGCCTTTCCAGCCAGACAAA
IL1B	AGCTGGAGAGTGTAGATCCCAA	ACGGGATGTTTTCTGCTTG
IL1RA	ATGGAGGGAAGATGTGCCTGTC	GTCCTGCTTTCTGTTCTCGCTC
IL6	ATCTGGATTCAATGAGGAGACTTG	TTGTACTCATCTGCACAGCTC
IL8	ACCGGAAGGAACCATCTCAC	GGCAAAACTGCACCTTCACAC
MKX	AGGCAATGCTGAACGGCTTAGC	TCACAGGATGGTGAACTGGGGT
MMP1	ACAACTGCCAAATGGGCTTG	AACAGCCCAGTACTTATTCCCTT
MMP3	CGTGCTTTGTCCTTTGATGCTG	GCGCCAAAAGTGCCTGTCTT
NANOG	CAGGAAATCGAGACCATCTTGGC	CTCTTTCTGCCTGCTTCTCCTTC
NGF	GGAGCGCAGCGAGTTTTGG	AGTGTGGCCAGGATAGAAAGC
OCT4	CCTGAAGCAGAAGAGGATCACC	AAAGCGGCAGATGGTCGTTTGG
PTGES2	CCTCTATGAGGCTGCTGACAAG	ATCACACGCAGCACGCCATACA
SCX	AGAACACCCAGCCCAAACAGAT	TCGCGGTCCTTGCTCAACTTT
SOX2	GCTACAGCATGATGCAGGACCA	TCTGCGAGCTGGTCATGGAGTT
TAC1	TAATGGGCAAGCGGGATGCTGA	CCATTAGTCCAACAAAGGAATCTG
TIMP1	AGACACCAGAGAACCCACCAT	TGACGAGGTCGGAATTGCAG
TNC	TGCTGAATGGAGACACGACC	AGGAACACAATCCATCCACCC
VEGFA	CATCCTGTGTGCCCCTGAT	TGATCCGCATAATCTGCATGG
RPL13A	GGCTTTCCTCCGCAAGCGGAT	GCAGCATACCTCGCACGGTCC
YWHAZ	ACCGTTACTTGGCTGAGGTTGC	CCCAGTCTGATAGGATGTGTTGG
B2M	CATTCCTGAAGCTGACAGCATTC	TGCTGGATGACGTGAGTAAACC
GAPDH	CATCACTGCCACCCAGAAGACTG	ATGCCAGTGAGCTTCCCGTTCAG
ACTB	CGCCGCCAGCTCACCATG	CACGATGGAGGGGAAGACGG
TBP	GCCACGCCAGCTTCGGAGAG	CCGCAGCAAACCGCTTGGGA
RPLP0	TGTGGGCTCCAAGCAGATGCA	GCAGCAGTTTCTCCAGAGCTGGG
18S rRNA	CTGCCCTATCAACTTTCGATGGTAG	CCGTTTCTCAGGCTCCCTCTC
