# Supplementary material for: Pulsed Electromagnetic Fields Modulate Inflammatory and Tenogenic Responses in Human Tenocytes: Insights from Acute and Prolonged Inflammation Models
Source: Cells. 2026 Aug 25;15(17):1529. doi: 10.3390/cells15171529 (PMC13565733; doi:10.3390/cells15171529)
Supplement: Supplementary file 1 [file cells-15-01529-s001.zip › Table_S4.pdf]

**Table S4. Effect sizes (η², % of total variation) and P values from two-way ANOVA for each outcome measure**

*η² expressed as % of total variation attributable to each source (IL-1β concentration, PEMF, IL-1β × PEMF interaction).*

| Section | Outcome | Figure | Source of Variation | η² (% total variation) | P value |
|---|---|---|---|---|---|
| **Proliferation — Acute model** | — | Fig 1A | **IL-1β** | 7.20 | 0.140 |
| | | | **PEMF** | 16.39 | **0.010** |
| | | | **Interaction** | 0.70 | 0.190 |
| **Proliferation — Prolonged model** | — | Fig 1B | **IL-1β** | 1.69 | 0.744 |
| | | | **PEMF** | 3.67 | 0.147 |
| | | | **Interaction** | 0.004 | 0.959 |
| **Gene expression — Acute model** | IL8 | Fig 3A | **IL-1β** | 85.92 | **0.005** |
| | | | **PEMF** | 0.50 | **0.007** |
| | | | **Interaction** | 1.78 | **0.008** |
| | IL6 | Fig 3B | **IL-1β** | 85.76 | **0.004** |
| | | | **PEMF** | 0.22 | 0.357 |
| | | | **Interaction** | 0.59 | 0.398 |
| | PTGES | Fig 3C | **IL-1β** | 52.50 | **0.006** |
| | | | **PEMF** | 1.49 | 0.588 |
| | | | **Interaction** | 1.29 | 0.679 |
| **Gene expression — Prolonged model** | CCL2 | Fig 3D | **IL-1β** | 7.54 | **0.007** |
| | | | **PEMF** | 78.77 | **0.0002** |
| | | | **Interaction** | 3.00 | **0.043** |
| | COX2 | Fig 3E | **IL-1β** | 77.31 | **0.002** |
| | | | **PEMF** | 4.61 | **0.012** |
| | | | **Interaction** | 5.16 | 0.103 |
| **ELISA — Acute model** | GM-CSF | Fig 4A | **IL-1β** | 83.90 | **0.0003** |
| | | | **PEMF** | 1.03 | **0.003** |
| | | | **Interaction** | 0.47 | **0.037** |
| | CCL2 | Fig 4B | **IL-1β** | 65.91 | **0.007** |
| | | | **PEMF** | 0.01 | 0.693 |
| | | | **Interaction** | 1.84 | **0.033** |
| | CCL5 | Fig 4C | **IL-1β** | $F(2,6) = 4.298$ | 0.070 |
| | | | **PEMF** | $F(1,3) = 5.182$ | 0.107 |
| | | | **Interaction** | $F(2,3) = 3.772$ | 0.152 |
| | IL6 | Fig 4D | **IL-1β** | 49.19 | **0.041** |
| | | | **PEMF** | 0.96 | 0.296 |
| | | | **Interaction** | 2.12 | 0.304 |
| | IL8 | Fig 4E | **IL-1β** | 77.39 | **0.003** |
| | | | **PEMF** | 1.36 | **0.023** |
| | | | **Interaction** | 1.96 | **0.004** |
| **ELISA — Prolonged model** | GM-CSF | Fig 4F | **IL-1β** | 41.68 | 0.092 |
| | | | **PEMF** | 3.18 | 0.136 |
| | | | **Interaction** | 2.08 | 0.129 |
| | CCL2 | Fig 4G | **IL-1β** | 74.14 | **0.018** |
| | | | **PEMF** | 1.44 | 0.081 |
| | | | **Interaction** | 1.32 | 0.073 |
| | CCL5 | Fig 4H | **IL-1β** | 40.10 | 0.137 |
| | | | **PEMF** | 0.41 | **0.028** |
| | | | **Interaction** | 0.41 | **0.028** |
| | IL6 | Fig 4I | **IL-1β** | 63.57 | **0.029** |
| | | | **PEMF** | 0.80 | 0.511 |
| | | | **Interaction** | 1.12 | 0.463 |
| | IL8 | Fig 4J | **IL-1β** | 47.72 | 0.090 |
| | | | **PEMF** | 0.53 | 0.461 |
| | | | **Interaction** | 0.60 | 0.426 |
| **MMP activity — Acute model** | — | Fig 5A | **IL-1β** | 66.90 | **0.002** |
| | | | **PEMF** | 0.81 | 0.631 |
| | | | **Interaction** | 2.21 | 0.549 |
| **MMP activity — Prolonged model** | — | Fig 5B | **IL-1β** | 80.22 | **0.013** |
| | | | **PEMF** | 0.22 | 0.457 |
| | | | **Interaction** | 0.55 | 0.287 |

*Note: P values <0.05 are shown in bold. For CCL5 (Fig. 4C), F values (numerator df, denominator df) are reported because a mixed-effects model was used.*
